# Redefining viability by cardiovascular magnetic resonance in acute ST-segment elevation myocardial infarction

**DOI:** 10.1038/s41598-017-15353-1

**Published:** 2017-11-07

**Authors:** Heerajnarain Bulluck, Stefania Rosmini, Amna Abdel-Gadir, Anish N. Bhuva, Thomas A. Treibel, Marianna Fontana, Daniel S. Knight, Sabrina Nordin, Alex Sirker, Anna S. Herrey, Charlotte Manisty, James C. Moon, Derek J. Hausenloy

**Affiliations:** 1 The Hatter Cardiovascular Institute, Institute of Cardiovascular Science, University College London, London, UK; 2 The National Institute of Health Research University College London Hospitals Biomedical Research Centre, London, UK; 3 Barts Heart Centre, St Bartholomew’s Hospital, London, UK; 4 National Amyloid Centre, Royal Free Hospital, London, UK; 5 Cardiovascular and Metabolic Disorders Program, Duke-National University of Singapore, Singapore, Singapore; 6 National Heart Research Institute Singapore, National Heart Centre Singapore, Singapore, Singapore; 7 Yong Loo Lin School of Medicine, National University Singapore, Singapore, Singapore

## Abstract

In chronic myocardial infarction (MI), segments with a transmural extent of infarct (TEI) of ≤50% are defined as being viable. However, in the acute phase of an ST-segment elevation myocardial infarction (STEMI), late gadolinium enhancement (LGE) has been demonstrated to overestimate MI size and TEI. We aimed to identify the optimal cut-off of TEI by cardiovascular magnetic resonance (CMR) for defining viability during the acute phase of an MI, using ≤50% TEI at follow-up as the reference standard. 40 STEMI patients reperfused by primary percutaneous coronary intervention (PPCI) underwent a CMR at 4 ± 2 days and 5 ± 2 months. The large majority of segments with 1–25%TEI and 26–50%TEI that were viable acutely were also viable at follow-up (59/59, 100% and 75/82, 96% viable respectively). 56/84(67%) segments with 51–75%TEI but only 4/63(6%) segments with 76–100%TEI were reclassified as viable at follow-up. TEI on the acute CMR scan had an area-under-the-curve of 0.87 (95% confidence interval of 0.82 to 0.91) and ≤75%TEI had a sensitivity of 98% but a specificity of 66% to predict viability at follow-up. Therefore, the optimal cut-off by CMR during the acute phase of an MI to predict viability was ≤75% TEI and this would have important implications for patients undergoing viability testing prior to revascularization during the acute phase.

## Introduction

Timely primary percutaneous coronary intervention (PPCI) is the revascularization strategy of choice for patients presenting with an acute ST-segment elevation myocardial infarction (STEMI). However, an increasing number of patients with an acute myocardial infarction (MI), such as those presenting late, and for those patients with multi-vessel coronary artery disease, urgent in-patient revascularization by coronary artery bypass graft surgery (CABG) may be needed. In those cases viability assessment in the acute phase of MI is desirable prior to subjecting these high-risk patients to a major procedure.

Viability can be assessed by cardiovascular magnetic resonance (CMR) using low-dose dobutamine stress or late gadolinium enhancement (LGE). Myocardial segments with a transmural extent of infarct (TEI) of ≤50% are normally accepted as being viable, and more likely to recover contractile function following revascularization in patients with stable coronary artery disease (CAD)^1,2^. However, in the acute phase of STEMI, LGE has been demonstrated to overestimate MI size and TEI^3–9^. Therefore, if a TEI of ≤50% is used to define viability by CMR in the acute phase of an MI, there is the possibility of over-estimating the number of non-viable segments, and missing potentially viable segments, when assessing patients prior to revascularization.

Therefore, the aim of the current study was to identify the optimal cut-off for TEI to define viability during the acute phase of an MI, which may be used when considering viability assessment for patients with recent acute MI requiring urgent in-patient revascularization.

## Results

Baseline characteristics of the 40 patients are listed in Table 1. The mean age of the STEMI patients was 59 ± 13 years old and 35/40 (88%) patient were male. The mean acute MI size was 27.4 ± 14.6 percent of the left ventricle (%LV) and the mean chronic MI size was 19.5 ± 10.5%LV. Out of 640 American Heart Association (AHA) segments the TEI were as follows: no LGE: 352 (55%); 1–25%TEI: 59 (9%); 26–50%TEI: 82 (13%); 51–75%TEI: 84 (13%); and 76–100%TEI: 63 (10%).

**Table 1:** Patient characteristics

	Number
Number of patients	40
Male (%)	35 (88%)
Age (age)	59 ± 13
Diabetes Mellitus	8 (20%)
Hypertension	14 (35%)
Smoking	12 (30%)
Dyslipidemia	14 (35%)
Chest pain onset to PPCI time (minutes)	267 [122–330]
Drug-eluting stent use (%)	38 (95%)
Infarct artery (%) LAD RCA Cx	24 (60%)14 (35%) 2 (5%)
TIMI flow Pre/ Post PPCI (%) 0 1 2 3	33 (83%)/ 1(3%) 0 (0%)/ 0 (0%) 3 (8%)/ 8 (20%) 4 (10%)/ 31 (78%)
CMR findings – acute/follow-up LV EDV/ml LV ESV/ml LV EF/% LV Mass/g MI size/ %LV MI size/ g MVO/%	172 ± 38/182 ± 49 90 ± 30/ 88 ± 38 49 ± 8/53 ± 10112 ± 35/104 ± 2627.4 ± 14.6/19.5 ± 10.520.2 ± 13.6/14.4 ± 9.4 26 (65%)/ 0

LAD: left anterior descending artery; RCA: right coronary artery; Cx: circumflex artery; TIMI: thrombolysis in myocardial infarction; CMR: cardiovascular magnetic resonance; LV: left ventricular; EDV: end diastolic volume; ESV: end systolic volume; EF: ejection fraction; MI: myocardial infarction; MVO: microvascular obstruction.

**Figure 1:**
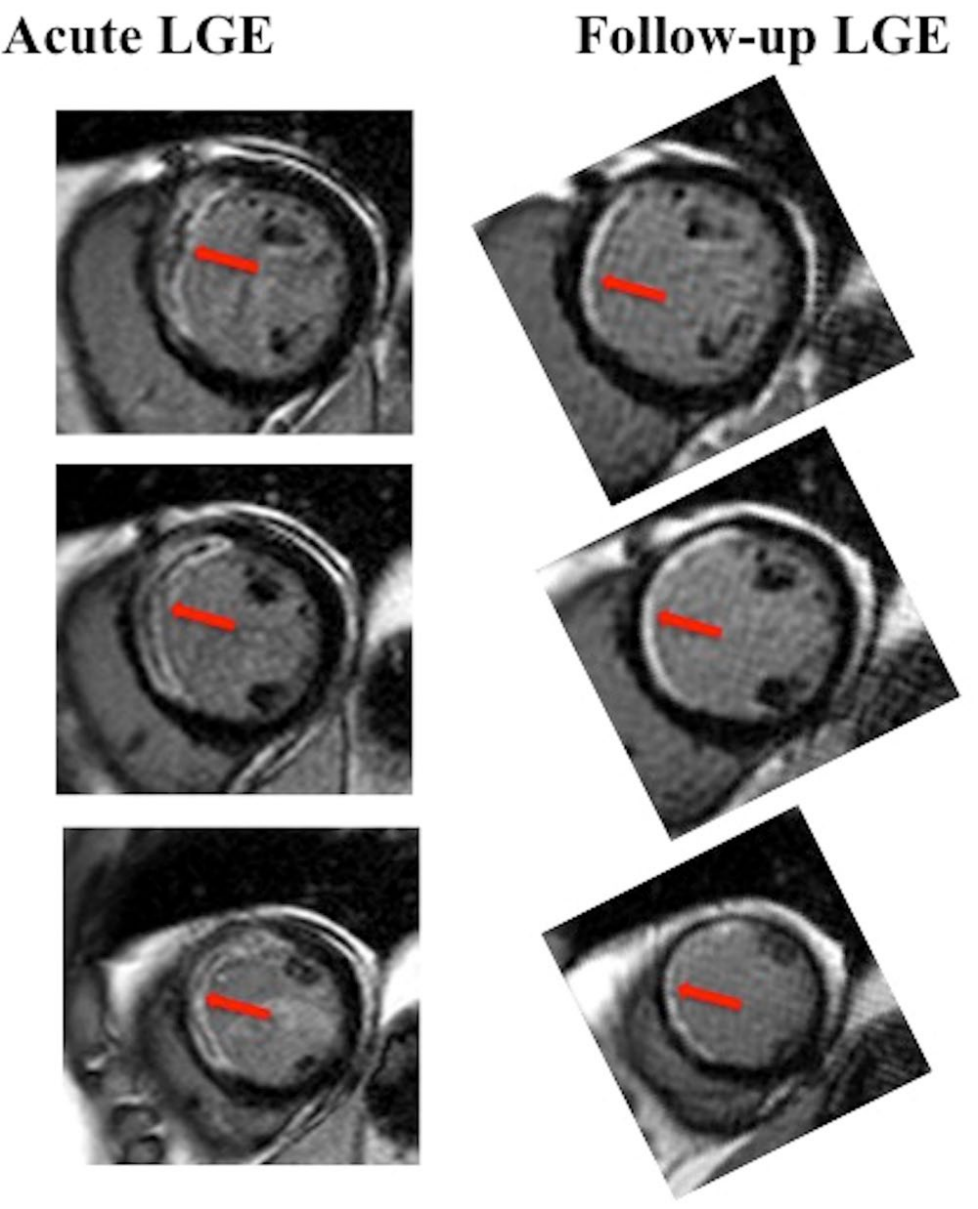
An example of MI size overestimation in the acute phase in a patient with an anterior STEMI. This patient presented with an anterior STEMI that was reperfused by PPCI. The left hand side panel depicts a large area of LGE with areas of MVO (surrounded by LGE) on the acute scan (red arrows) and the corresponding LGE on the follow-up scan (red arrows) on the right hand side panel. The area of non-infarcted myocardium is present on the epicardial side of the MVO on the acute scan and is more obvious on the follow-up scan. The follow-up scan shows the resolution of the MVO and thinning of the LGE area. The follow-up LGE images were rotated to match the acute scan orientation.

Figure 1 shows an example of a patient with anterior STEMI reperfused by PPCI and a large area of LGE with areas of microvascular obstruction (MVO) on the acute scan (red arrows). The corresponding LGE on the follow-up scan (red arrows) demonstrates a significant reduction in MI size.

### TEI on the acute scan and viability at follow-up

**Figure 2:**
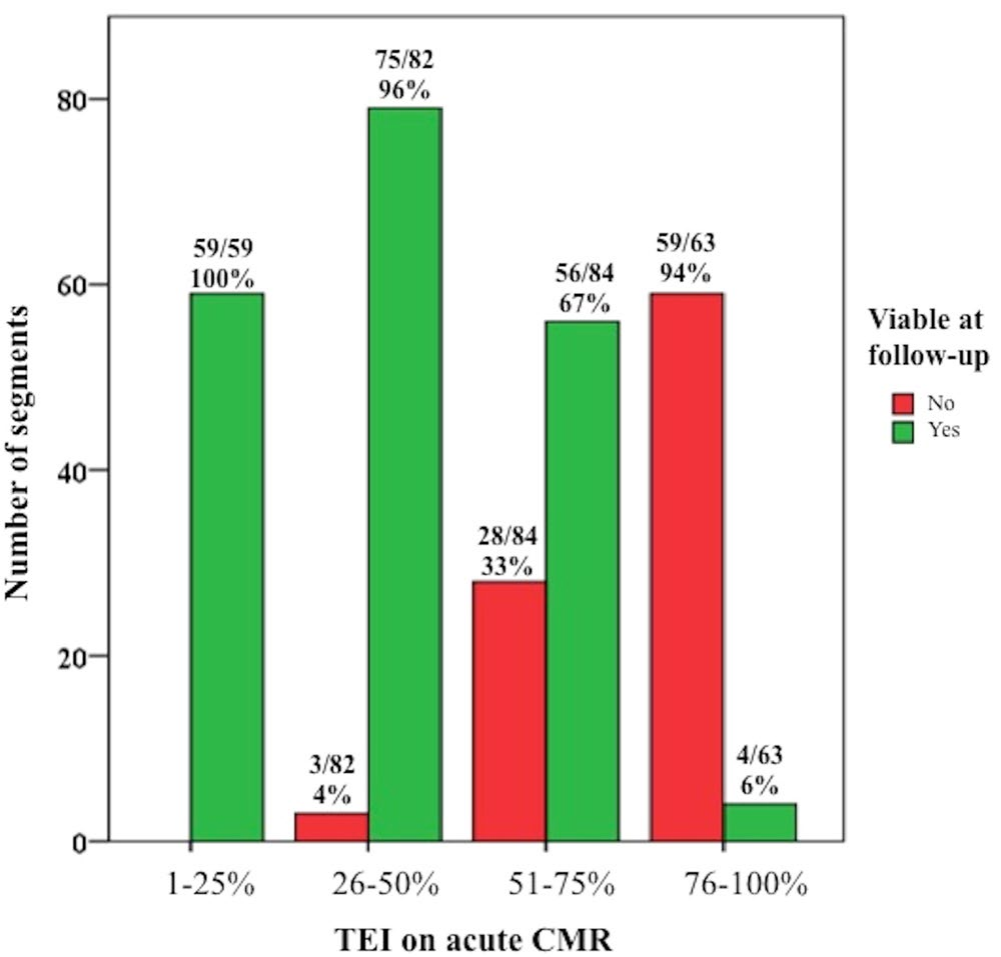
Distribution of TEI segments on the acute CMR scan and subsequent viability at follow-up. This figure shows the distribution of myocardial segments with LGE in the 4 groups of TEI on the acute scan, and subdivided as viable or not at follow-up.

288/640 (45%) myocardial segments had LGE on the acute CMR scan. For segments in the 1–25%TEI and 26–50%TEI groups, a large proportion of segments (59/59, 100% and 75/82, 96%) that were viable acutely, were also viable at follow-up. 56/84 (67%) segments in the 51–75%TEI group were reclassified as viable on the follow-up scan (Fig. 2). A further 4/63 (6%) segments from >76–100%TEI group were also found to be viable at follow-up. Clustered Receiver Operating Characteristic (ROC) curve analysis showed that the 4 TEI classification on the acute scan had an area-under-the-curve (AUC) of 0.87 (95% CI 0.82–0.91) to predict viability at follow-up. Segments with ≤75%TEI on the acute CMR scan had a sensitivity of 98% but a specificity of 66% to predict viability at follow-up.

77/288 (27%) segments with LGE had MVO. All these segments had at least ≥26%TEI. Among those segments with MVO in the 26–50%TEI group, 7/8 (88%) were viable at follow-up; among those in the 51–75%TEI group, 12/23 (52%) were viable at follow-up. However, those in the 76–100%TEI group, only 1/46 (2%) were viable at follow-up.

Among segments with LGE, ROC curve analysis showed that presence MVO had an AUC of 0.77 (95% CI 0.70–0.83) to predict viability (sensitivity of 94% but a specificity of 63%).

### TEI on the acute scan and segmental wall motion recovery at follow-up

**Figure 3:**
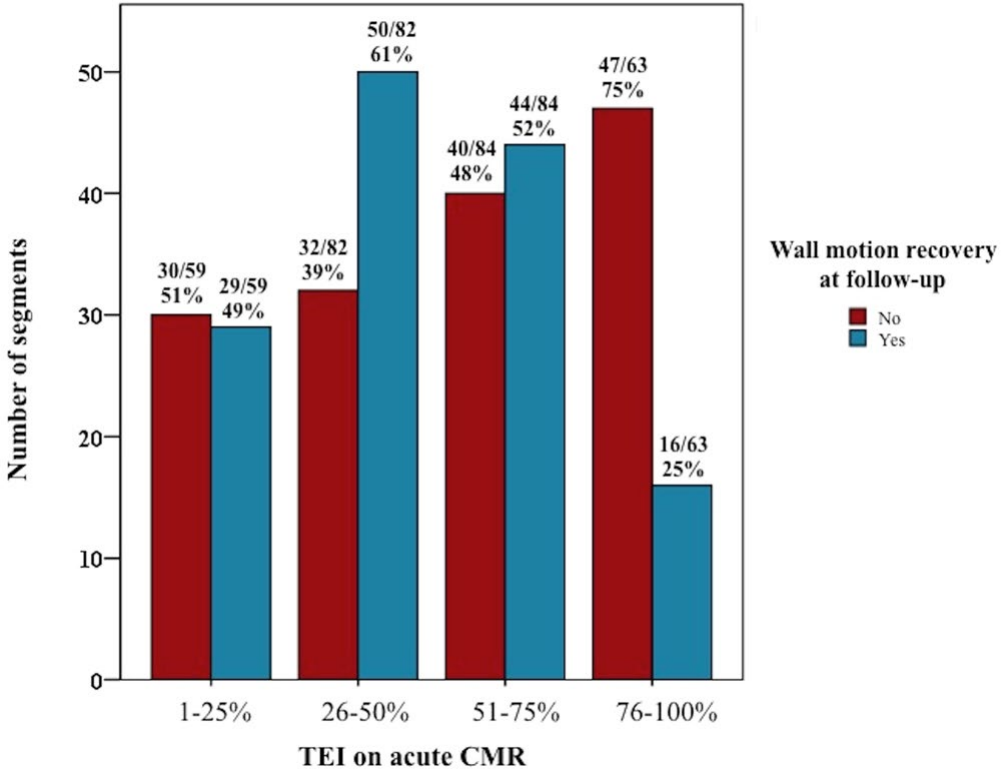
Distribution of TEI segments on the acute scan and wall motion recovery. This figure shows the distribution of segment with LGE in the 4 groups of TEI on the acute scan and subdivided by whether there was a segmental wall motion recovery at follow-up or not.

Figure 3 shows the distribution of segments with LGE in the 4 groups of TEI on the acute scan, and subdivided by whether there was a segmental wall motion recovery at follow-up or not. Segments with 76–100%TEI were less likely to have wall motion recovery compared to the other 3 TEI groups (P < 0.001). Clustered ROC curve analysis showed that TEI on the acute scan had an AUC of 0.72 (95% CI 0.64–0.80) to predict wall motion recovery at follow-up.

### Relationship between wall motion and viability

Among segments without LGE on the acute scan, 41/352 (12%) had regional wall motion abnormality that resolved completely at follow-up. When considering segments with LGE only on the acute scan (n = 288), the clustered ROC curve analysis for regional wall motion abnormality on the acute scan to predict viability at follow-up had an area under the curve of 0.42 (95%CI 0.36–0.49).

**Figure 4:**
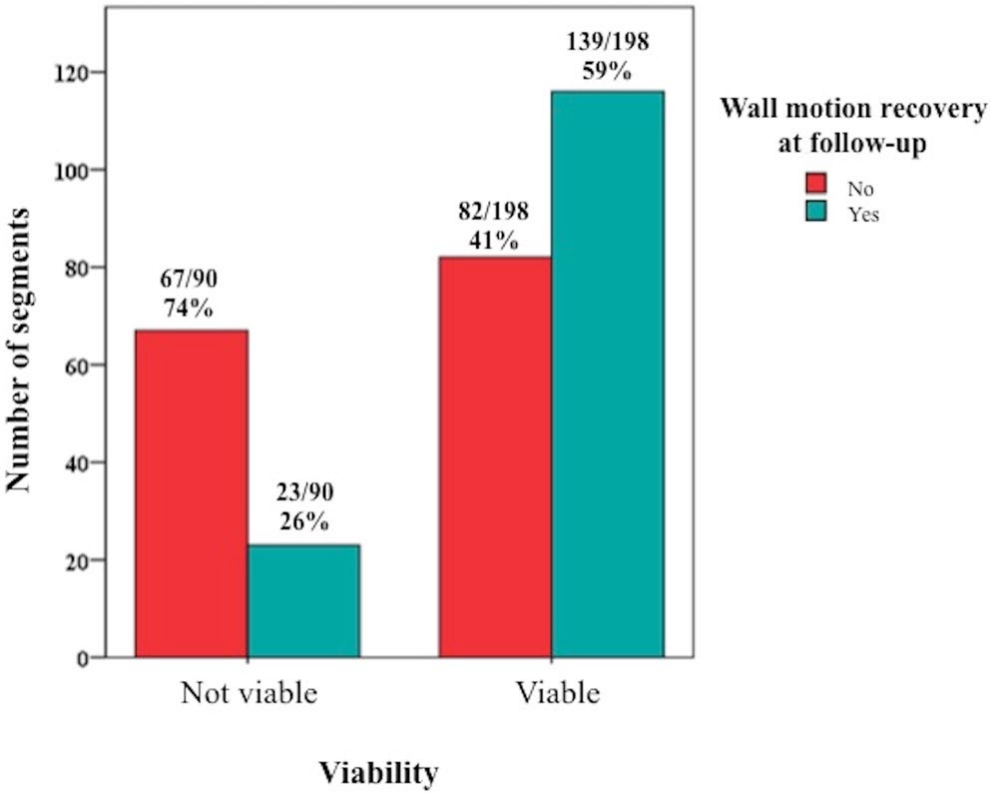
Viability and wall motion recovery. This figure shows the distribution of viable and non-viable segments and their subsequent wall motion recovery.

139/198 (59%) of the viable segments improved wall motion compared to 23/90 (26%) of non-viable segments showed an improvement in wall motion, P < 0.001 (Fig. 4). Wall motion recovery was significantly associated with viability after adjusting for within-patient interaction between segments and wall motion (R^2^ 0.44, P < 0.001).

## Discussion

The main findings from our study are that two thirds of segments with a TEI of 51–75% on the acute CMR scan were reclassified as having a TEI ≤50% and being viable at follow-up. Furthermore, more than half of the segments with MVO in the 51–75% TEI group were reclassified as being viable at follow-up. A TEI of ≤75% on the acute scan was the optimal TEI to predict viability at follow-up with a very high sensitivity of 98%, but a specificity of 66%. Wall motion score on the acute scan in segments with LGE was not a good predictor of viability at follow-up. As expected, viable segments were more likely to have wall motion recovery.

A ≤50%TEI by LGE is the conventional cut-off used for defining viability in patients with stable CAD, and has been validated in the setting of revascularization by CABG or PCI^1,2^. Kim *et al*.^1^ showed that in 90% of segments with 51–75% TEI did not improve wall motion in 41 patients after revascularization. Selvanayagam *et al*.^2^ also showed that a significant proportion of patients with ≤50%TEI improved regional wall motion in 52 patients undergoing CABG surgery. It is already well recognized that LGE overestimates MI size in the first few days following an acute STEMI^3–8^, and therefore using ≤50%TEI to define viability may be inaccurate in the acute setting. A previous small study of 12 patients showed that a ≤50% TEI on the acute CMR had a lower sensitivity (88%) than the ≤50% TEI on the follow-up scan (94%) to predict viability using positron emission tomography with 18F-fluorodeoxyglucose performed at 1 year as the reference standard^10^. However the number of patients in that study was very small. To our knowledge, no other studies have formally assessed the TEI in the acute setting that would correspond to a viable segment at follow-up. We have shown that segments with up to ≤75%TEI on the acute scan should be considered viable in cases when viability assessment is performed in the acute setting using LGE.

Several studies have already shown that a significant regression in MI size occurs between the acute and follow-up scans^3–7,11^ and this was confirmed in our study. Acute MI size is also dynamic and reduces in size within the first week^3–6,12^. Whether LGE also delineates reversibly injured myocardium is a topic of ongoing debate. It may be possible for LGE to delineate the infarcted as well as the salvaged myocardium when LGE images are acquired too early (<8 minutes from contrast injection)^13^. However, in most clinical studies LGE images are acquired after 10 to 15 minutes and therefore this may not be a major contributing factor for the overestimation of acute MI size. Lastly, depending of the contrast dose, timing of acquisition of LGE, the sequence used and the method used for LGE quantification, inclusion of the peri-infarct grey zone as part of the MI size would also contribute to the overestimation of the acute MI size.

Several studies have shown that the TEI on the acute CMR scan is a good predictor of segmental wall motion recovery^8,14,15^. We also showed that a significant proportion of segments with up to 75%TEI recovered wall motion compared to those segments with >75%TEI. Although segments with wall motion recovery were more likely to be viable, wall motion score alone on the acute scan was not a good test to predict viability. This is likely due to the fact that, firstly, myocardial stunning within the first week of an acute MI was prone to affect viable segments as well, and secondly, the inaccurate assessment of wall motion in predominantly non-viable segments due to tethering of surrounding segments that were viable could have led to the false appearance of an improvement in wall motion. Extracellular volume fraction (ECV) of the MI zone in the acute setting has previously been shown to complement LGE to predict wall motion recovery at follow-up^16^ and to predict chronic MI size^9^. Unlike LGE that dichotomizes the myocardium as either “myocardial necrosis/scar” or “normal”, ECV has the added advantage to quantify the severity of the myocardial injury in the MI zone. However, interpreting areas of MVO and intramyocardial hemorrhage on the ECV maps remains a challenge and more work is needed before parametric mapping can be translated in the clinical setting to assess viability in acute MI.

There are several limitations in our study. Our sample size was small. We only included STEMI patients post-PPCI, and did not include patients who were scheduled for viability assessment in the acute phase of an acute MI prior to revascularization. However, we indirectly showed that segments that would be classified as non-viable on the acute scan could be viable if the scan had been performed a few months later. Although, intuitively, the results of our study is expected as MI size is overestimated in the acute phase^3–7^, further studies including both STEMI as well as non-STEMI patients undergoing viability assessment in the acute phase prior to revascularization are required to confirm our findings.

In conclusion, we have shown that the optimal cut-off by CMR during the acute phase of an MI to predict viability was ≤75% TEI. Two third of segments with 50–75% TEI on the acute scan were reclassified as having ≤50% TEI at follow-up. This would have important implications for those patients with an acute MI undergoing viability testing by CMR prior to revascularization as an in-patient and these findings need to be confirmed in future studies.

## Methods

### Study Population

Patients from a previously reported cohort were included^17–23^. In brief, the London-Harrow Research Ethics Committee approved this study. 50 STEMI patients were prospectively recruited from August 2013 to July 2014 following informed consent. 48 patients completed the first CMR at 4 ± 2 days post-PPCI and 40 patients had a follow-up scan at 5 ± 2 months. The 40 patients with paired acute and follow-up CMR scans were analyzed for this study. All research-related procedures were performed in accordance with the local guidelines and regulations.

#### Late gadolinium enhancement acquisition

All CMR scans were performed on a 1.5 Tesla scanner (Magnetom Avanto, Siemens Medical Solutions) using a 32-channel phased-array cardiac coil. Full LV coverage LGE imaging was acquired using either a standard segmented ‘fast low-angle shot’ two-dimensional inversion recovery gradient echo sequence or a respiratory motion-corrected, free-breathing single shot steady state free precession averaged inversion recovery LGE sequence^24^ at 10 minutes after the injection of 0.1 mmol/kg of Gadoterate meglumine (marketed as Dotarem, Guerbet S.A., Paris, France). The acquisition parameters of the LGE sequences have been previously reported^17–21^. For both LGE sequences, the inversion times were progressively optimized to null the normal remote myocardium (typical values 360 to 440ms). Matching sequences with the same parameters and gadolinium contrast dose and timing of LGE acquisition were used for the follow-up scan.

### Imaging analysis

All imaging analysis was performed using CVI42 software (Version 5.1.2[303], Calgary, Canada).

#### Quantification of MI size

The endocardial and epicardial borders were manually delineated on all the LGE short-axis LV images. A reference region of interest was drawn in the remote normal myocardium using the automated option available on CVI42 to minimise variability (with minimal manual adjustment when needed). MI size was quantified using a signal intensity threshold of 5 standard deviations (SD) above the normal remote myocardium^25,26^ and expressed both as %LV and in grams. TEI was also quantified objectively by averaging the values from 100 chords from each short axis slice to obtain the mean transmural extent of LGE for each of the 16 segments and expressed in as a continuous variable and then objectively classified into groups of “no LGE”, “1–25%TEI”, “26–50%TEI”, “51–75% TEI” and “76–100%TEI” as per the 16-segment AHA model.

A cut-off value of ≤50% TEI on the follow-up scan was used to define viability^1,2^. The same cut-off was used to define viability on the acute scan in the absence on any established cut-off of TEI to define viability in the acute setting.

Segmental wall motion on the short axis cine images were visually scored by 2 experienced investigators (both the first and second authors with 3 years experience in CMR) as “0” for normal; “1” for mild/moderate hypokinesis; “2” for severe hypokinesis; “3” for akinesis and “4” dyskinesis^1^ and displayed as per the 16-segment AHA classification^27^. Wall motion recovery was defined as an improvement in wall motion score by 1.

### Statistical analysis

SPSS version 22 (IBM Corporation, Illinois, US) and Stata 12.1(StataCorp LP, Texas, USA) was used for all statistical analysis. Shapiro-Wilk Test was used to assess for normality. Continuous data were expressed as mean ± SD or median (interquartile range) and compared with paired student t test/Wilcoxon signed rank test or unpaired student t test/Mann Whitney U test where appropriate. To take into consideration potential within-subject interaction of segments with LGE and subsequent viability at follow-up, clustered ROC analysis was conducted to assess the performance of TEI groups to predict viability and regional wall motion recovery at follow-up. A linear mixed-effects model was used to assess the relationship between viability at follow-up and regional wall motion recovery. All statistical tests were two-tailed, and P < 0.05 was considered statistically significant.
